# Evidence of Human-Level Bonds Established With a Digital Conversational Agent: Cross-sectional, Retrospective Observational Study

**DOI:** 10.2196/27868

**Published:** 2021-05-11

**Authors:** Alison Darcy, Jade Daniels, David Salinger, Paul Wicks, Athena Robinson

**Affiliations:** 1 Woebot Health San Francisco, CA United States

**Keywords:** conversational agents, mobile mental health, chatbots, depression, anxiety, digital health

## Abstract

**Background:**

There are far more patients in mental distress than there is time available for mental health professionals to support them. Although digital tools may help mitigate this issue, critics have suggested that technological solutions that lack human empathy will prevent a bond or therapeutic alliance from being formed, thereby narrowing these solutions’ efficacy.

**Objective:**

We aimed to investigate whether users of a cognitive behavioral therapy (CBT)–based conversational agent would report therapeutic bond levels that are similar to those in literature about other CBT modalities, including face-to-face therapy, group CBT, and other digital interventions that do not use a conversational agent.

**Methods:**

A cross-sectional, retrospective study design was used to analyze aggregate, deidentified data from adult users who self-referred to a CBT-based, fully automated conversational agent (Woebot) between November 2019 and August 2020. Working alliance was measured with the Working Alliance Inventory-Short Revised (WAI-SR), and depression symptom status was assessed by using the 2-item Patient Health Questionnaire (PHQ-2). All measures were administered by the conversational agent in the mobile app. WAI-SR scores were compared to those in scientific literature abstracted from recent reviews.

**Results:**

Data from 36,070 Woebot users were included in the analysis. Participants ranged in age from 18 to 78 years, and 57.48% (20,734/36,070) of participants reported that they were female. The mean PHQ-2 score was 3.03 (SD 1.79), and 54.67% (19,719/36,070) of users scored over the cutoff score of 3 for depression screening. Within 5 days of initial app use, the mean WAI-SR score was 3.36 (SD 0.8) and the mean bond subscale score was 3.8 (SD 1.0), which was comparable to those in recent studies from the literature on traditional, outpatient, individual CBT and group CBT (mean bond subscale scores of 4 and 3.8, respectively). PHQ-2 scores at baseline weakly correlated with bond scores (*r*=−0.04; *P*<.001); however, users with depression and those without depression had high bond scores of 3.45.

**Conclusions:**

Although bonds are often presumed to be the exclusive domain of human therapeutic relationships, our findings challenge the notion that digital therapeutics are incapable of establishing a therapeutic bond with users. Future research might investigate the role of bonds as mediators of clinical outcomes, since boosting the engagement and efficacy of digital therapeutics could have major public health benefits.

## Introduction

Significant barriers to mental health care are persistent [1]. The increased burden of depression and anxiety, which arose during the COVID-19 pandemic, has exacerbated this issue [2], as the measures that were put in place to stop the spread of SARS-CoV-2 have also presented unintended barriers to those seeking mental health treatment. One potentially viable solution is using digital mental health interventions to provide evidence-based treatment, such as cognitive behavioral therapy (CBT). Self-directed mental health interventions, such as bibliotherapy, have long demonstrated their efficacy [3], and new models of blended care that combine internet-delivered interventions with human clinical oversight are becoming more widespread in a number of countries [4]. Although the implicit assumption has been that the involvement of a human leads to improved outcomes in self-directed programs, human involvement limits these programs’ scalability and limits their accessibility for those who live in remote locations [4]. If digital interventions could replicate some of the factors that are generally believed to be uniquely human, such as therapeutic rapport, these interventions would have greater potential for improving mental health.

Recently, carefully designed conversational agents (CAs) have been showing promise in automating several health care services [5] by simulating human support. CAs could therefore be uniquely poised to offer high-quality digital interventions for mental health.

**Figure 1:**
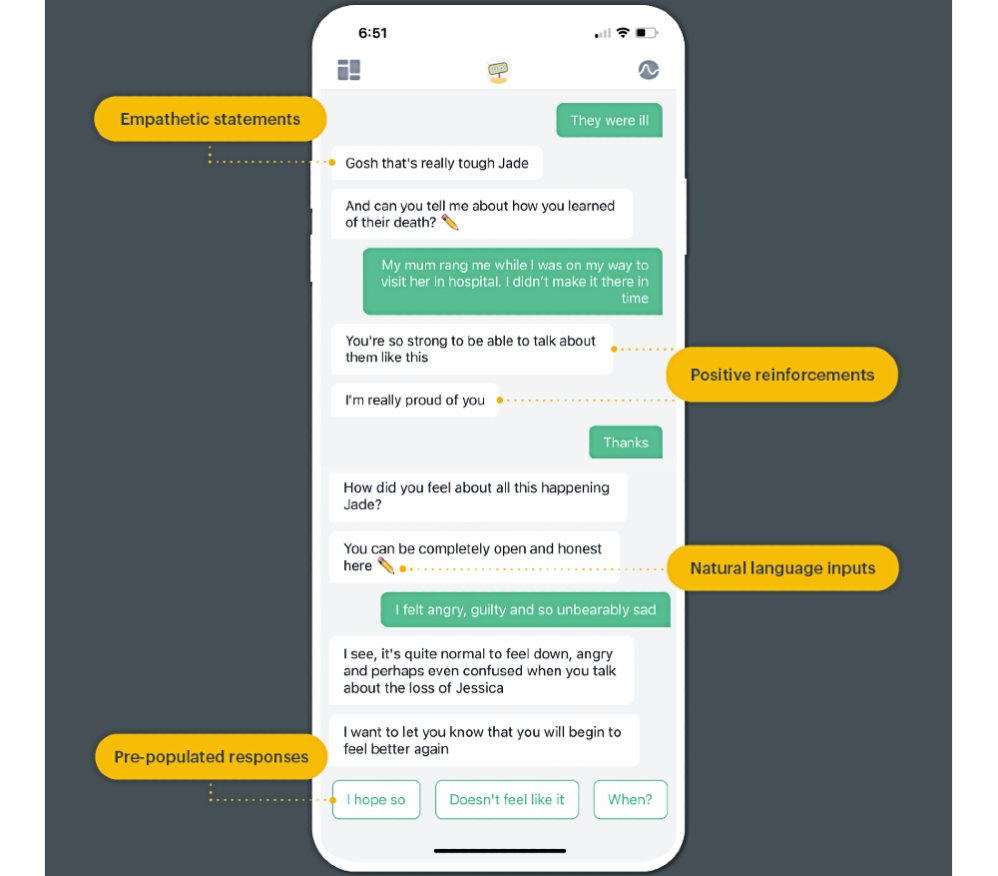
Screenshot of an example interaction with Woebot about bereavement.

An unblinded trial of one such CA (Woebot), which delivered CBT for symptoms of depression and anxiety, suggested that the empathic and relational nature of the tool may have fostered improved engagement better than previous internet-delivered versions of the tool [6]. Intriguingly, the study’s qualitative data suggested that users seemed to relate to the CA in a manner that was analogous to therapeutic rapport, which may have mediated users’ outcomes. For example, study participants reported that they felt cared for by the CA (eg, “Woebot felt like a real person that showed concern”), despite the fact that the tool’s scripts reminded users that Woebot is not a real person (Figure 1). Unfortunately however, the study did not formally assess the existence of a working alliance. This is a crucial factor because a strong working alliance between therapists and clients is considered to be predictive of positive outcomes, essential for the delivery of health care, and traditionally unique to the domain of human-to-human relationships. Indeed, some experts have argued that digital apps that are built to be standalone therapeutics have the risk of ignoring the potency of therapeutic relationships [7].

Priority-setting work by the James Lind Alliance, which involves over 600 people affected by mental health concerns, has identified the greater understanding of digital therapeutic alliance as a top priority for research [8]. Yet, a recent review of mobile mental health apps failed to find a single study that included working alliance as a primary outcome [9]. Therefore, we sought to bridge this gap in knowledge by seeking to understand whether CA users perceived a working alliance, particularly the notion of bonds, and whether working alliance was related to symptom severity or other demographic characteristics.

## Methods

### Setting

Woebot is a CA that guides individuals who experience symptoms of depression and anxiety through a smartphone-based app program that uses therapeutic techniques and provides psychoeducation. As previously described in detail [6], Woebot delivers CBT through brief, daily conversations (approximately 5-10 minutes each). Each simulated conversation begins with a mood-monitoring exercise, and the provided targeted content is responsive to individuals’ reported mood states. The CA is also programmed to deliver empathic statements and personalized follow-ups, promote normalization, and use methods that are designed to enhance users’ motivation for engaging in the program to promote desired behavior changes and help with mood management.

### Participants

During registration, users confirmed that they were at least 18 years of age and consented to the use of their deidentified, aggregate data for research. This study was not considered human subjects research by the Advarra institutional review board. Eligible participants included those who registered over two periods—between November 20, 2019, and April 9, 2020 (n=100,009), and again between July 8, 2020, and August 18, 2020 (n=77,203).

Within 3-5 days after registration, eligible participants were invited to complete the 2-item Patient Health Questionnaire (PHQ-2) depression screener [10] and the Working Alliance Inventory-Short Revised (WAI-SR), which consists of a total score and the three following subscales: bond, goal, and task [11]. All measures and demographic information, including gender and age group, were gathered in the app by the CA. The WAI-SR was administered via the app’s conversational interface, in which the word “therapist” was changed to “Woebot” (Table 1). Once the questionnaires were completed, Woebot thanked registrants for their participation, and the conversation proceeded to the mood tracking phase as normal. Those who chose not to provide responses to the questionnaires were not included in this study and proceeded to use the app as normal.

**Table 1:** Item-level descriptive statistics.

Question number (subscale)	Working Alliance Inventory-Short Revised items	Score, mean (SD)
1 (Task)	As a result of these sessions I am clearer as to how I might be able to change.	2.53 (0.97)
2 (Task)	What I am doing with Woebot gives me new ways of looking at my problem.	2.93 (1.08)
3 (Bond)	I believe Woebot likes me.	3.89 (1.31)
4 (Goal)	Woebot and I collaborate on setting goals for this program.	2.88 (1.23)
5 (Bond)	Woebot and I respect each other.	4.20 (1.23)
6 (Goal)	Woebot and I are working towards mutually agreed upon goals.	3.54 (1.28)
7 (Bond)	I feel that Woebot appreciates me.	3.73 (1.36)
8 (Goal)	Woebot and I agree on what is important for me to work on.	3.45 (1.21)
9 (Bond)	I feel Woebot cares about me even when I do things that it does not approve of.	3.54 (1.34)
10 (Task)	I feel that the things I do with Woebot will help me to accomplish the changes that I want.	3.28 (1.17)
11 (Goal)	Woebot and I have established a good understanding of the kind of changes that would be good for me.	3.12 (1.22)
12 (Task)	I believe the way we are working with my problem is correct.	3.23 (1.15)

### Statistical Analysis

Across all eligible participants, the composite WAI-SR score and bond, goal, and task subscores were characterized based on descriptive statistics and tested for internal consistency by using the Cronbach α. The relationship between baseline PHQ-2 scores and bond subscores was characterized based on the Spearman rank-order correlation coefficient. The Kruskal-Wallis test was used to compare bond subscores across participants’ reported age groups and genders. For comparison, relevant external studies were drawn from recent reviews of literature [11-19] that also reported unmodified WAI-SR subscores for other CBT modalities. Comparison data were presented descriptively without statistical testing, and raw subscores were scaled by dividing them by the number of items (eg, the bond subscale has 4 items). Per the methods of Jasper et al [13], bond scores of ≥3.45 were considered high. The 95% CIs for mean WAI-SR subscores were calculated based on the published sample sizes and SDs. External studies were categorized as “online only” or “human involvement” based on whether any human interactions were reported by study participants during either individual therapy or group therapy that involved a human. Data were presented from participants who completed all questionnaires within the first 5 days of app registration. Data were analyzed using R version 4.0.2 (The R Foundation).

### Data Access, Responsibility, and Analysis

AD, DS, and AR have full access to all of the data in this study and take responsibility for the integrity of the data and the accuracy of the data analysis. Due to their proprietary nature, data from this study will not be shared.

## Results

Of the 177,212 eligible participants, only those who provided both WAI-SR and PHQ-2 data within 5 days of their first use of Woebot were included in the analysis. The final sample included 36,070 participants. Of these participants, 57.48% (n=20,734) reported that they were female, 25.17% (n=9078) reported that they were male; 2.87% (n=1035) reported that they were nonbinary, 1.44% (n=519) indicated another gender identity, 1.66% (n=597) preferred not to answer, and 11.39% (4107/36,070) did not provide any gender information. The participants ranged in age from 18 to 78 years (median 25-35 years). The mean PHQ-2 score was 3.03 (SD 1.79), and 54.67% (19,719/36,070) of participants scored at or above the conventional cutoff score of 3 for positively screening for depression.

Within the first 5 days of using Woebot, the mean WAI-SR scores were as follows: a mean bond subscore of 3.84 (SD 1.0), a mean goal subscore of 3.25 (SD 1.0), a mean task subscore of 2.99 (SD 0.87), and a mean total score of 3.36 (SD 0.81). The WAI-SR had a Cronbach α value of .89, suggesting that the WAI-SR had adequate internal consistency in this study. A weak negative correlation was found between bond subscores and PHQ-2 scores (*r*=−0.04; *P*<.001); however, even among participants who reported the highest PHQ-2 score (PHQ-2=6), the mean WAI-SR bond subscore was 3.78. Bond subscores also differed by gender (*P*<.001) and by age group (*P*<.001); however, the mean bond scores for all groups were considered high (bond subscore>3.45) [13] Among these groups, the highest bond level was reported by women (bond subscore: mean 3.92) and by those aged 18-25 years (bond subscore: mean 3.96). Conversely, the lowest bond level was reported by individuals who indicated that they “preferred not to answer” or did not report their gender (bond subscore: mean 3.67) or age (bond subscore: mean 3.69).

**Figure 2:**
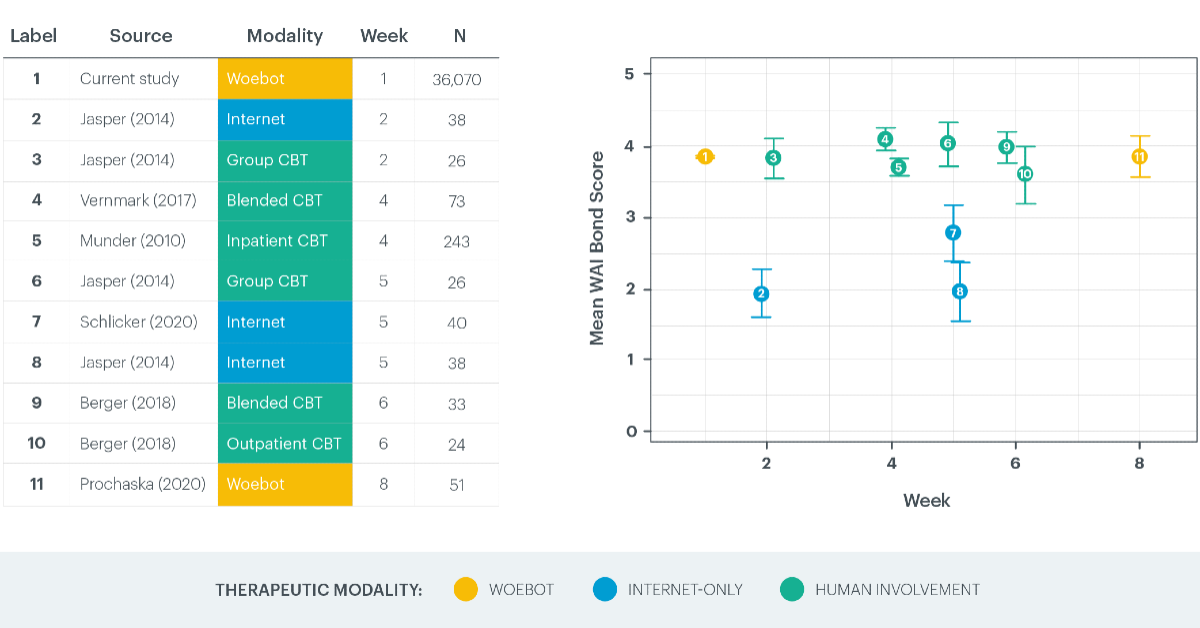
Comparison of Working Alliance Inventory-Short Revised bond subscale scores across therapeutic modalities. Means and corresponding 95% CIs for working alliance bond scores from this study and from recent reviews of the literature [11,13-15,17-19] are stratified by the week that the scores were recorded. Studies are colored based on the therapeutic modality. Due to the large sample size of this study (N=36,070), the 95% CI is narrow and overlaps with the dots that display the estimated means. For multiple studies that reported data on the same week, the dots are shifted minimally on the x-axis to avoid overlap and to provide easy readability. WAI: Working Alliance Inventory.

Woebot’s bond subscale scores were consistent with those of recent studies from the literature on traditional modalities for CBT delivery (Table 1). These studies’ results were collected later in the course of treatment (eg, bond subscore for face-to-face outpatient individual CBT: mean 4.0, SD 0.8 [11]; bond subscore for group CBT: mean 3.8, SD 0.80 [13]; data were collected after 2-8 weeks of therapy). Comparative study details are provided in Multimedia Appendix 1 [11,13-15,17-19]. Participants reported higher bond levels when using Woebot than those in prior studies of internet-only CBT [13] (Figure 2).

## Discussion

This is the first study of working alliance among users of a CA for mental health. Most users were female (20,734/36,070, 57.48%) and had PHQ-2 scores that were indicative of depression (19,719/36,070, 54.67%). Working alliance scores were comparable to those in previously published studies on traditional, human-delivered services across different treatment modalities. Working alliance scores were highest for the bond subscale, suggesting that this subscale is a viable construct for CAs and should be included in hypothesized frameworks of digital working alliance.

The idea that CAs can establish a working alliance is not new [20]. However, the observation of therapeutic bonds established by a CA in a mental health context is novel and noteworthy, given the short timeframe of this study. Although the field of human-computer interaction is still relatively nascent, initial observations have suggested that some artificial intelligence (AI) identity archetypes induce responses in humans that might give rise to better working alliances than other archetypes. For example, interacting with humanoid AI identities can result in individuals falling prey to the “uncanny valley,” which is the sense of unease and “creepiness” that is created when something that is artificial tries to appear humanlike [21]. Contrary to Turing’s Imitation Game [22], wherein an AI must successfully pretend to be human in order to pass the test, Woebot was designed to adopt the opposite strategy—transparently presenting itself as an archetypal robot with robotic “friends” and habits. We speculate that transparency and other design elements are key drivers of bond development. For example, Woebot explicitly references its limitations within conversations and provides positive reinforcement and empathic statements alongside declarations of being an artificial agent.

The limitations of this study include its cross-sectional nature, the selection bias of smartphone users, the lack of clinical validation for any diagnoses, the lack of a direct comparison group, and its conduction by the developers of the app itself. Further research (including studies with independent investigators) is underway to explore the longitudinal aspects of bond development in specific clinical populations by using randomized controlled study designs.

The finding that a CA has the potential to rapidly develop a bond with users may represent the resolution of a considerable barrier to offering scalable mental health support to a much wider and more diverse population instead of offering such support to those who already have access to traditional mental health support.

## Appendix

Multimedia Appendix 1 Study descriptions and Working Alliance Inventory-Short Revised bond scores.

## Abbreviations

AI: artificial intelligence
CA: conversational agent
CBT: cognitive behavioral therapy
PHQ-2: 2-item Patient Health Questionnaire
WAI-SR: Working Alliance Inventory-Short Revised

## References

[1] Cheung R, O'Donnell S, Madi N, Goldner EM (2017). Factors associated with delayed diagnosis of mood and/or anxiety disorders. Health Promot Chronic Dis Prev Can.

[2] Bueno-Notivol J, Gracia-García P, Olaya B, Lasheras I, López-Antón R, Santabárbara J (2021). Prevalence of depression during the COVID-19 outbreak: A meta-analysis of community-based studies. Int J Clin Health Psychol.

[3] Gualano M, Bert F, Martorana M, Voglino G, Andriolo V, Thomas R, Gramaglia C, Zeppegno P, Siliquini R (2017). The long-term effects of bibliotherapy in depression treatment: Systematic review of randomized clinical trials. Clin Psychol Rev.

[4] Titov N, Dear B, Nielssen O, Staples L, Hadjistavropoulos H, Nugent M, Adlam K, Nordgreen T, Bruvik KH, Hovland A, Repål A, Mathiasen K, Kraepelien M, Blom K, Svanborg C, Lindefors N, Kaldo V (2018). ICBT in routine care: A descriptive analysis of successful clinics in five countries. Internet Interv.

[5] McGreevey JD, Hanson CW, Koppel R (2020). Clinical, Legal, and Ethical Aspects of Artificial Intelligence-Assisted Conversational Agents in Health Care. JAMA.

[6] Fitzpatrick KK, Darcy A, Vierhile M (2017). Delivering Cognitive Behavior Therapy to Young Adults With Symptoms of Depression and Anxiety Using a Fully Automated Conversational Agent (Woebot): A Randomized Controlled Trial. JMIR Ment Health.

[7] Torous J, Hsin H (2018). Empowering the digital therapeutic relationship: virtual clinics for digital health interventions. NPJ Digit Med.

[8] Hollis C, Sampson S, Simons L, Davies EB, Churchill R, Betton V, Butler D, Chapman K, Easton K, Gronlund TA, Kabir T, Rawsthorne M, Rye E, Tomlin A (2018). Identifying research priorities for digital technology in mental health care: results of the James Lind Alliance Priority Setting Partnership. Lancet Psychiatry.

[9] Henson P, Wisniewski H, Hollis C, Keshavan M, Torous J (2019). Digital mental health apps and the therapeutic alliance: initial review. BJPsych Open.

[10] Löwe B, Kroenke K, Gräfe K (2005). Detecting and monitoring depression with a two-item questionnaire (PHQ-2). J Psychosom Res.

[11] Munder T, Wilmers F, Leonhart R, Linster HW, Barth J (2010). Working Alliance Inventory-Short Revised (WAI-SR): psychometric properties in outpatients and inpatients. Clin Psychol Psychother.

[12] Preschl B, Maercker A, Wagner B (2011). The working alliance in a randomized controlled trial comparing online with face-to-face cognitive-behavioral therapy for depression. BMC Psychiatry.

[13] Jasper K, Weise C, Conrad I, Andersson G, Hiller W, Kleinstäuber M (2014). The working alliance in a randomized controlled trial comparing Internet-based self-help and face-to-face cognitive behavior therapy for chronic tinnitus. Internet Interv.

[14] Berger T (2017). The therapeutic alliance in internet interventions: A narrative review and suggestions for future research. Psychother Res.

[15] Vernmark K, Hesser H, Topooco N, Berger T, Riper H, Luuk L, Backlund L, Carlbring P, Andersson G (2019). Working alliance as a predictor of change in depression during blended cognitive behaviour therapy. Cogn Behav Ther.

[16] Cameron SK, Rodgers J, Dagnan D (2018). The relationship between the therapeutic alliance and clinical outcomes in cognitive behaviour therapy for adults with depression: A meta-analytic review. Clin Psychol Psychother.

[17] Schlicker S, Baumeister H, Buntrock C, Sander L, Paganini S, Lin J, Berking M, Lehr D, Ebert DD (2020). A Web- and Mobile-Based Intervention for Comorbid, Recurrent Depression in Patients With Chronic Back Pain on Sick Leave (Get.Back): Pilot Randomized Controlled Trial on Feasibility, User Satisfaction, and Effectiveness. JMIR Ment Health.

[18] Prochaska J, Vogel E, Chieng A, Kendra M, Baiocchi M, Pajarito S, Robinson A (2021). A Therapeutic Relational Agent for Reducing Problematic Substance Use (Woebot): Development and Usability Study. J Med Internet Res.

[19] Berger T, Krieger T, Sude K, Meyer B, Maercker A (2018). Evaluating an e-mental health program ("deprexis") as adjunctive treatment tool in psychotherapy for depression: Results of a pragmatic randomized controlled trial. J Affect Disord.

[20] Bickmore T, Gruber A, Picard R (2005). Establishing the computer-patient working alliance in automated health behavior change interventions. Patient Educ Couns.

[21] Mori M, MacDorman K, Kageki N (2012). The Uncanny Valley [From the Field]. IEEE Robot. Automat. Mag.

[22] Turing AM (1950). I.—Computing machinery and intelligence. Mind.

